# Isolating Neural Correlates of the Pacemaker for Food Anticipation

**DOI:** 10.1371/journal.pone.0036117

**Published:** 2012-04-27

**Authors:** Ian David Blum, Elaine Waddington Lamont, Trevor Rodrigues, Alfonso Abizaid

**Affiliations:** Department of Neuroscience, Carleton University, Ottawa, Ontario, Canada; Vanderbilt University, United States of America

## Abstract

Mice fed a single daily meal at intervals within the circadian range exhibit food anticipatory activity. Previous investigations strongly suggest that this behaviour is regulated by a circadian pacemaker entrained to the timing of fasting/refeeding. The neural correlate(s) of this pacemaker, the food entrainable oscillator (FEO), whether found in a neural network or a single locus, remain unknown. This study used a canonical property of circadian pacemakers, the ability to continue oscillating after removal of the entraining stimulus, to isolate activation within the neural correlates of food entrainable oscillator from all other mechanisms driving food anticipatory activity. It was hypothesized that continued anticipatory activation of central nuclei, after restricted feeding and a return to *ad libitum* feeding, would elucidate a neural representation of the signaling circuits responsible for the timekeeping component of the food entrainable oscillator. Animals were entrained to a temporally constrained meal then placed back on *ad libitum* feeding for several days until food anticipatory activity was abolished. Activation of nuclei throughout the brain was quantified using stereological analysis of c-FOS expressing cells and compared against both *ad libitum* fed and food entrained controls. Several hypothalamic and brainstem nuclei remained activated at the previous time of food anticipation, implicating them in the timekeeping mechanism necessary to track previous meal presentation. This study also provides a proof of concept for an experimental paradigm useful to further investigate the anatomical and molecular substrates of the FEO.

## Introduction

All mammals exhibit circadian (daily) patterns of behaviour and physiology regulated by a complex network of endogenous clocks and coordinated by the master pacemaker in the suprachiasmatic nucleus of the hypothalamus (SCN) in order to phase align to the solar day [1], [2]. Since zeitgeber (German, from *zeit* ‘time’ and *geber* ‘giver’) information may not necessarily reach every tissue in the body directly, there is a necessity for a hierarchical organization of information processing from the master pacemaker to central and peripheral cellular clock networks [3]. Furthermore, it has been experimentally demonstrated that there is an SCN-independent circadian system responsible for the rhythmic behavioural, tissue level, cellular, and molecular processes in response to patterns of food intake [4]. Although the exact mechanism(s) responsible for the timekeeping properties of this pacemaker are presently unknown [5], this system is generally referred to as the food entrainable oscillator (FEO). The behavioural output of this FEO exhibits canonical properties of circadian pacemaker control including: the requirement of a zeitgeber for initial entrainment, a limited range of entrainment falling within a circadian timeframe (∼22–29 hrs) [6], [7], [8], persistence of precisely timed oscillations for several cycles during sustained food deprivation [9], [10], [11], and transient rather than immediate resetting after a change in meal time [12]. Although many physiological processes undergo temporal realignment under this type of feeding schedule, the output of the FEO typically used as a behavioral measure is food anticipatory locomotor activity (FAA), which has a robust, high amplitude oscillation that is easily recorded automatically with minimal disturbance to the subject.

Accumulated evidence has lead several groups to propose that the FEO may be a distributed system of coupled central and peripheral oscillators, unlike the anatomically circumscribed system found in the SCN [2], [13], [14], [15]. Indeed, one of the key differences between light-entrained circadian rhythms and the food entrainable system relies on a fundamental difference between the sensory mechanisms that provide their respective zeitgeber inputs. The SCN is entrained by light impinging on the retinal ganglion cells [16], [17], [18]. In contrast, the food-entrainable system relies on the integration of food-derived cues that act at a myriad of peripheral and central sites throughout the nervous system, using various independent mechanisms [19].

In determining the brain regions that contribute to a distributed FEO, a major challenge is to dissociate neural activity of the FEO from those of the metabolic homeostat and the appetitive/affective centers, all of which interact and converge to produce behavioural FAA under restricted feeding (RF) conditions. In order meet this challenge, this study investigated the neural correlates of the FEO under conditions that minimize the secondary effects of restricted feeding, those that do not rely on circadian-driven anticipation of food, but rather are activated in response to metabolic challenge. This was accomplished by entraining animals to a daytime meal then eliminating the influence of a negative metabolic state by allowing three days of *ad libitum* feeding, a timeframe shown to be sufficient to abolish FAA in rats [7], [8], [9], [20] and further confirmed in these mice. Returning animals to *ad libitum* feeding does not disrupt the phase or function of the FEO, as previous studies have shown that FAA can be reinstated by a 24-hour fast, even after many, many days of *ad libitum* feeding [4], [21]. However, allowing *ad libitum* feeding reduced any neural activation due to the homeostatic, appetitive, and affective drives observed either during RF or a subsequent fasting period, thus allowing an examination of brain regions involved in the maintenance of food-related circadian rhythms, without the confound of a negative metabolic state.

## Materials and Methods

### Ethics Statement

All manipulations were conducted under the guidelines set forth by the Canadian Council on Animal Care and approved by Carleton University's Animal Care Committee.

### Animals

Twenty-four CD1 adult male mice (Charles River Laboratories, St. Constant, QC; 20–25 gm upon arrival) were single-housed in standard mouse cages (Nalgene) and received *ad libitum* chow and water in a temperature and humidity controlled vivarium with a 12-hour light-dark (L∶D) cycle starting at 8:00 AM (Zeitgeber Time 0 (ZT0)). An additional 8 mice were housed in metabolic chambers (TSE Systems, Inc., Chesterfield, MO, USA) under identical environmental conditions in order to automatically assess rhythms of feeding and drinking.

### Procedure

#### Behavioral measures

**Figure 1:**
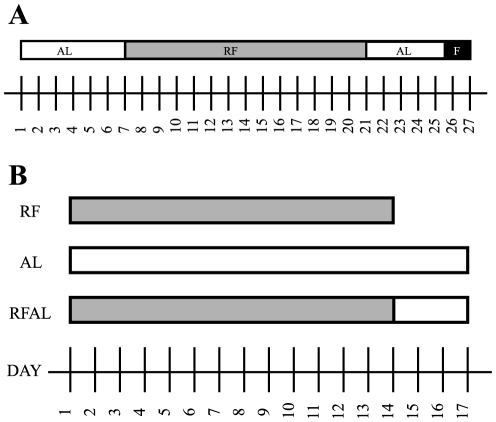
Food Restriction Paradigm. **A,** typical restricted feeding paradigm used in mice; including *ad libitum* baseline (AL), restricted feeding with food available only for a short temporal window during the light phase leading to entrainment (RF), *ad libitum* refeeding to abolish FAA (AL), and finally a two day fast to verify circadian entrainment (F). **B,** groups and treatments employed in this study. Restricted feeding occurred between ZT 4 and 8 during RF with all body weight and food consumption measures taken at ZT8.

In the first experiment, mice were randomly assigned to one of three groups: *Ad libitum* fed (AL, n = 8), restricted feeding schedule (RF, n = 8) or restricted feeding schedule then *ad libitum* fed (RF+AL, n = 8). Whereas inn the metabolic chambers, 3 mice were assigned to the AL condition, and 5 were placed in the RF+AL condition. Animals in the AL condition (negative control) received free access to food throughout the study. Animals in the RF group (positive control) had restricted access to food to 4 hours during the day (from ZT 4-ZT8), and were sacrificed after 14 days at approximately ZT2–3, prior to food access. Finally, mice in the RF+AL group were exposed to the same restricted feeding protocol as those in the RF group, but then were allowed *ad libitum* access to food for three days in order to abolish FAA before tissue collection at the time of previous meal anticipation (See **Figure 1**). In our second study, RF+AL animals in metabolic chambers had 7 days of scheduled feeding before returning to *ad libitum* food access for 3 days.

#### Locomotor activity

Locomotor activity was measured in the animals' home cage using a Superflex HC infrared locomotor activity monitoring module connected to a computer (Accuscan Instruments, Columbus, OH). Ambulatory activity data, defined by beam breaks, were continually collected in 6 minute bins over 24 hours using Fusion HC v1.5 software (Accuscan Instruments, Columbus, OH). Several measures were calculated including food anticipatory activity (activity in the 2 hours preceding the presentation of scheduled meal), total daily activity, nocturnal activity, and nocturnality (defined as nocturnal activity as a percentage of total 24 hr activity). Daily food consumption and body weight were recorded at ZT8 using an SI-2002 digital scale (Denver Instruments, Bohemia, NY). The data was divided into each feeding condition for analysis 1) entrainment, days 1 through 14 and 2) *ad libitum* feeding, days 15–17. Repeated Measures ANOVAs (α = 0.05) and planned comparisons between experimental groups (AL vs. RF vs. RFAL) were performed using PASW statistics v17.0 (SPSS Worldwide, Chicago, IL) to analyze the effect of experimental group, time and any interaction between these two factors.

#### c-FOS Immunocytochemistry

On the last day of behavioural recordings between ZT3–4, mice were injected with an overdose of sodium pentobarbital. A syringe with a 23G needle was then used to collect a blood sample from the atrium, followed by intra-cardial perfusion of 100 ml of saline (0.9%), then 200 ml of paraformaldehyde 4%. The animals were then decapitated and brains were collected and post-fixed overnight in the same fixative. Brains were then transferred into vials containing a 30% sucrose solution until sliced in their entirety using a Cryotome FSE cryostat (Thermo Fisher Scientific, Waltham, MA). Sections were divided into 4 serial sets of 60 µm slices and were immediately stored in a cryo-protectant solution at −20°C until processed for immunocytochemistry [22].

To examine neuronal activation, sections were processed for cFOS immunocytochemistry as described previously [23]. In short, brain sections were rinsed with shaking 5×5 min in cold phosphate buffer (0.1 M PB; pH 7.3), incubated for fifteen minutes in a quenching solution (1% H_2_O_2_ in 0.1 M PB), rinsed three times in cold 0.1 M PB, followed by a blocking solution (0.3% Triton-X detergent and bovine serum albumen at 0.01 g/mL in 0.1 M PB) for thirty minutes at room temperature (RT), and finally c-FOS (Anti-c-FOS Ab-5 4–17 Rabbit pAb, Calbiochem) diluted 1∶20 000 in a solution identical to the blocking solution and agitated for forty-eight hours at 4°C. After incubation with the primary antibody, sections were rinsed (5×5 min. in 0.1 M PB) and incubated for one hour with a an anti-rabbit secondary antibody (Biotinylated goat Anti-Rabbit IgG H+L 1∶250; Vector Labs) diluted in 0.1 M PB containing Triton-X and BSA. After incubation with the secondary antibody, the sections were rinsed (3×5 min. in 0.1 M PB) at RT for one hour with the Avidin-Biotinylated Enzyme Complex (ABC Complex; Vector Labs). Sections were again rinsed (3×5 min. in 0.1 M PB) and incubated in a solution of 3,3′-diaminobenzidine (DAB; 1 mL per 16 mL of buffer; Sigma) and Cobalt Chloride (600 µL per 17 mL of 0.1 M PB), and developed for 4 min. using 25 µL of a 0.1% H_2_O_2_ solution. After final rinsing, sections were wet-mounted onto gelatin-coated slides, dehydrated in three consecutive series of alcohols and coverslipped with Permount (Fisher).

#### Quantification of immunocytochemistry

Sections were examined using an Olympus BX51 brightfield microscope (Olympus Canada, Markham, ON) and associated digital capture device DVC-2000C (DVC Company, Austin, TX) connected to a desktop computer with Windows XP operating system (Microsoft Corp., Redmond, WA). Three-dimensional stereological reconstructions using every fourth brain slice across the entire extent of each ROI were used to estimate cell density and analyzed to determine changes in c-FOS IR. Due to various stochastic factors including: missing sections for some animals or failure to meet an appropriate Gunderson's coefficient of error (described below), the first 5 regions (out of the eight total), passing such criterion per group, per brain region, were retained for analysis using the optical fractionator probe within Stereo Investigator v 9 (MBF bioscience, Williston, VT). Regions of interest (ROI), chosen *a priori* based on previous investigations observing changes in c-FOS under RF [24], [25], [26], [27], [28], [29] were identified using a mouse brain atlas (see **Table 1** for a full list of regions, their associated map coordinates, and the sampling parameters used). Cell counting was performed using sampling parameters determined to be sufficient to produce a Gunderson's coefficient of error (GCE, m = 1) less than 0.1. The GCE (m = 1) has been determined to be the most appropriate coefficient of error estimate for all non-homogeneous biological tissues including cell populations within the brain [30], [31]. Furthermore, a histogram analysis of the distribution of cells throughout the extent of each slice was performed while assessing other sampling parameters to ensure that no “dead zones” caused by sub-optimal antibody penetration were apparent. This analysis revealed an even distribution of cells along the z-axis when the uppermost and lowermost 4 µm of tissue were excluded. Sampling parameters for each ROI (listed in Table 1) were chosen to ensure that the estimated cell counts did not include undue stochastic variability (as represented by the GCE), and once determined, were held constant across all three experimental groups. Each ROI was outlined digitally (4× magnification) using the software provided while referring to the mouse brain atlas [32] for comparison. This information was then analyzed to create a volumetric estimate of each ROI measure in µm^3^. Each nucleus sampled was individually examined at high magnification using an oil immersion lens (60× magnification) and the uppermost tip of c-FOS positive nuclei were labeled with a digital marker. The software provided used both the planar (XY) and depth (Z) information recorded for each marker in the analysis of cell population estimates thereby creating a three dimensional model of the distribution of cells within each section from which an estimate of the total population throughout the entire extent of the ROI is created. Cellular population estimates were then divided by the estimated volume in order to create an estimate of the density of cells expressing c-FOS such that comparisons between animals take into account the intrinsic variability between the shape and size of ROIs. For each ROI, differences in mean cell densities between groups were analyzed using one-way ANOVAs and followed by planned comparison (LSD) tests with an α = 0.05 critical level. These analyses were conducted using PASW statistics v 17.0 software (SPSS Worldwide, Chicago, IL).

**Table 1:** Regions of interest for stereological analysis of c-FOS positive cell densities.

Region of Interest	Abbr.	Sampled∶Total Volume	*Stereotaxic Coordinates*
Arcuate Nucleus	ARC	1∶4	1.22 mm–2.46 mm posterior
Basolateral Amygdala	BLA	1∶8	1.06 mm–1.94 mm posterior
Oval Nucleus of the Bed Nucleus of the Stria Terminalis*	BNSTov	1∶4	0.38 mm–0.14 mm anterior
Central Amygdala	CeA	1∶8	1.2 mm–1.70 mm posterior
Cingulate Gyrus	Cg1	1∶8	2.34 mm–0.22 mm anterior
Dentate Gyrus	DG	1∶4	1.34 mm–2.46 mm posterior
Dorsomedial Hypothalamus	DMH	1∶8	1∶8
Dorsal Raphé	DR	1∶8	4.04 mm–4.96 mm posterior
Locus Coeruleus	LC	1∶4	5.34 mm–5.80 mm posterior
Lateral Hypothalamus	LH	1∶8	0.34 mm–2.80 mm posterior
Nucleus Accumbens Core	NAcbC	1∶8	1.94 mm–0.74 mm anterior
Nucleus Accumbens Shell	NAcbSH	1∶8	1.94 mm–0.74 mm anterior
Nucleus of the Tractus Solaris	NTS	1∶4	6.42 mm–7.92 mm posterior
Parabrachial Nucleus	PB	1∶4	5.02 mm–5.68 mm posterior
Prefrontal Cortex	PFC	1∶8	3.08 mm–1.54 mm anterior
Paraventricular Hypothalamic Nucleus	PVN	1∶4	0.58 mm–1.22 mm posterior
Suprachiasmatic Nucleus	SCN	1∶4	0.22 mm–0.82 mm posterior
Ventromedial Hypothalamus	VMH	1∶4	1.06 mm–2.06 mm posterior
Ventral Tegmental Area	VTA	1∶8	2.92 mm–3.88 mm posterior

Coordinates displayed relative to bregma according to *The Mouse Brain in Stereotaxic Coordinates* [32] except for * taken from *Basic organization of projections from the oval and fusiform nuclei of the bed nuclei of the stria terminalis in adult rat brain* [58]. The counting frame was kept at 100 µm^2^ for all regions and the grid size was altered based on initial parameter estimates.

#### Quantification of PER2 positive cells in the stomach

Paraformaldhyde-fixed stomachs were embedded in paraffin and sliced at a thickness of 15 µm using a vertical microtome, (n = 6 for each group), then mounted individually on glass slides using a heated waterbath. Each slice was then dewaxed and rehydrated before processing for DAB immunochemistry using an anti-PER2 primary antibody (1∶3,000, Alpha Diagnostic Inc., San Antonio, TX). PER2 expression was quantified by calculating the relative optical density (ROD). To this end, an Olympus BX51 brightfield microscope (Olympus Canada, Markham, ON) and associated digital capture device DVC-2000C (DVC Company, Austin, TX) were used to observe stomach slices at 100× magnification while holding the exposure time (25 ms) and irradiance (full power with ND6 and ND25 filters engaged) constant. Images were digitized using the *image capture* function within Stereo Investigator v 9 (MBF bioscience, Williston, VT) and saved as 16bit grayscale TIFF images. Relative optical density was calculated using the *measure* function within Image J v 1.4 (National Institute of Health, USA). For each slice, the polygon selection tool was used to outline both the oxyntic mucosa and a blank area of the slide in order to measure the *mean grey values* of each. This returned a value that represents the average of all grey values (with a possible value of 1 for black to 256 for white). In order to determine the optical density of each region, these values were then transformed to fit a linear standard curve where 100 represented the highest optical density and 0 represented the lowest [(100-(Y/256*100)), where Y represents the mean grey value]. Finally, the ROD was calculated by subtracting the optical density of the background from that of the oxyntic mucosa. Values were then analyzed using PASW statistics v 17.0 (SPSS Worldwide, Chicago, IL) to perform a one-way ANOVA with *posthoc* LSD test (α = 0.05) and any group differences between experimental conditions were observed.

#### Ghrelin Enzyme Linked Immunoassay (ELISA)

Blood samples obtained at the time of sacrifice (about 400 µl each) were collected in EDTA laced tubes containing 5 µl of 0.1 M HCl and 5 µl of p-hydroxymercuribenzoic acid (PHMB: Fluka) at a concentration of 100 nM to slow the denaturing of the active form of ghrelin. Samples were then centrifuged (300 rpm for 5 min. at 4°C) to separate plasma. Plasma was stored at −80°C until processing for ELISA. Samples from 3 mice in the AL group, 2 in the RF group and 1 in the RFAL group were lost during collection. Plasma content of active (acylated) ghrelin from the remaining samples was determined using a commercially available ELISA kit (EZRGRA-90K, Millipore). All samples were assayed in duplicate and differences were evaluated using a one-way between groups ANOVA. Interassay variability was less than 10%.

## Results

**Figure 2:**
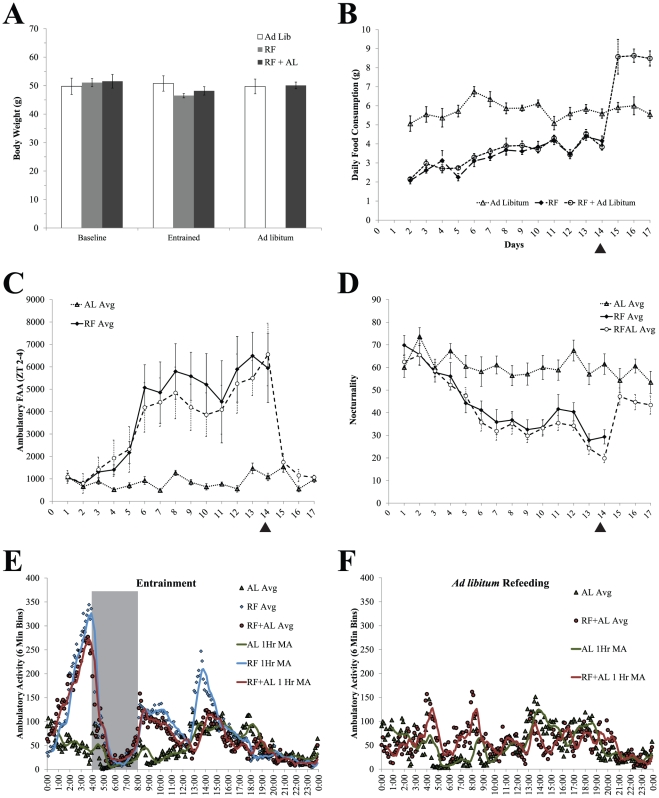
Food Intake, Body Weight, and Behaviour. **A,** average body weights for each group at the end of each feeding treatment. **B,** average daily food consumption for each group across days. Note the sharp peak in rebound feeding after a return to ad libitum. However, both the initial reduction and subsequent rebound in feeding had no effect on body weight across conditions suggesting internal metabolic compensation/protection against food restriction. **C,** average daily values for ambulatory activity during the time of meal anticipation (ZT4–8). Note that FAA develops slowly over several days as the animals entrain to their new feeding schedule but sharply declines back to baseline values on day 15 after being returned to *ad libitum* feeding. **D,** nocturnality scores for animals during RF and subsequent AL feeding. Nocturnality is defined as the total ambulatory activity at night divided by total daily activity. **E,** average waveforms of all animals during the last week of restricted feeding. Note the sharp increase of FAA prior to meal presentation. **F,** average waveform during the third day of *ad libitum* feeding, The anticipatory peak and the increase in night time activity was completely abolished after a return to *ad libitum* feeding, although daytime activity did appear to remain slightly higher than AL controls. Note: 1 hr MA represents a smoothed curve where the moving average, the average score for all data points within the hour surrounding each time point, was plotted. Gray bars indicate the time of food availability and black triangles indicate the first day of ad libitum feeding.

Scheduled feeding was associated with changes in food intake and body weight. Mice in the RF groups consumed less food during the time they had access to it than control ad lib fed mice (*F*_(2,24)_ = 4.972, *p*<0.05, Fisher's LSD). However, animals in the RFAL group ate more than ad lib controls once they were again allowed ad lib access (*F*_(1,14)_ = 34.892, *p*<0.05, LSD; see **Figure 2**). Furthermore, there were no overall significant differences in body weight between groups during or after the entrainment period (*p*>0.05).

**Figure 3:**
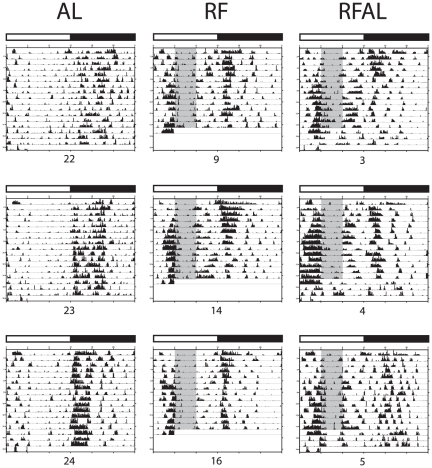
Representative actograms demonstrating beam break activity across the entire experiment. Note both the quantity and temporal structure of FAA during the restricted feeding paradigm and its loss after the RFAL animals are placed back on *ad libitum feeding*. Gray bars indicate the time of food access for the RF and RFAL groups.

As expected, restricted feeding schedules produced significant alterations in daily patterns of locomotor activity. Specifically, anticipatory locomotor activity was elevated in the RF and RFAL groups relative to AL controls (*F*_(6,120)_ = 4.125, *p*.<0.05 LSD; see **Figure 2**). Nocturnality was similarly reduced in the RF and RFAL groups as compared to controls (*F*_(6,120)_ = 0.240, *p* = 0.788, *p.*<0.05, LSD; see **Figure 2**). During the subsequent *ad libitum* feeding period, there was a significant change in FAA but not nocturnality across days (*F*_(2,26)_ = 10.229, *p* = 0.004 and *F*_(2,26)_ = 1.639, *p* = 0.218 respectively) according to the Greenhouse-Geisser criterion. However, both the expression of FAA and the nocturnality score were not significantly different from the AL group by the third day (*F*_(1,13)_ = 1.351, *p* = 0.266 and *F*_(1,13)_ = 4.292, *p* = 0.059 respectively; see **Figure 2**). These effects, as well as the temporal structure of activity across days are also visible in the representative actograms provided **(** **Figure 3** **)**.

**Figure 4:**
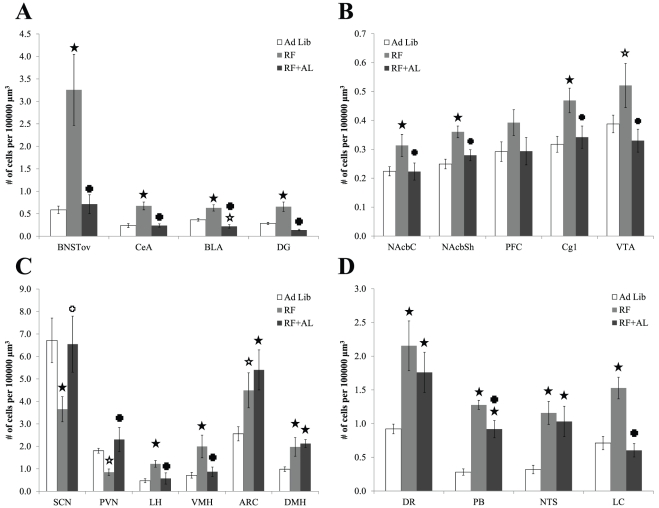
Mean c-FOS cell density (+/−SE). **A,** Affective system nuclei. **B,** Appetitive system nuclei **C,** Hypothalamic nuclei **D,** Brainstem nuclei. Stars indicates a significant difference from AL controls (*p*<0.05), crosses indicate significant difference from RF group (*p*<0.05). Hollow symbols denote differences approaching significance (*p*<0.1).

**Figure 5:**
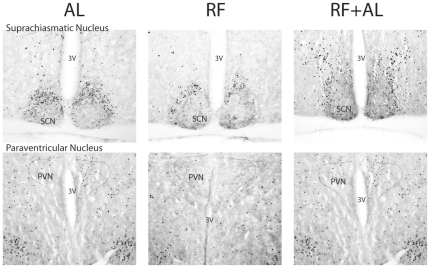
Representative images showing suppression of c-FOS expression in the PVN and SCN. Notice that c-FOS appears preferentially suppressed in the core nucleus of the SCN but not the shell, whereas the PVN is suppressed in both the parvocellular and magnocellular nuclei.

**Figure 6:**
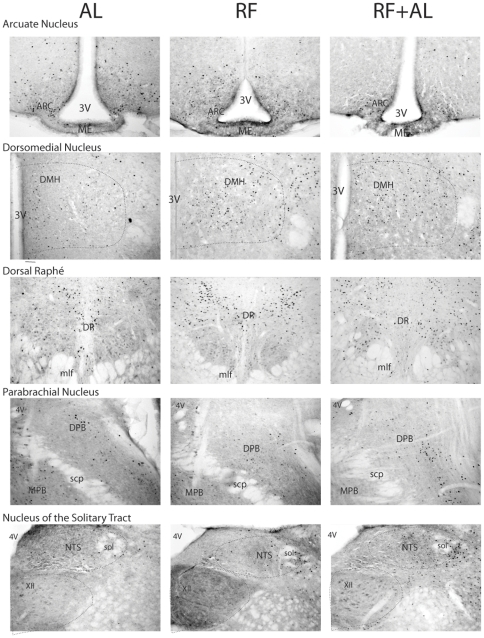
Representative images showing c-FOS expression in candidate nuclei involved in the pacemaking component of the FEO. Note the increased c-FOS expression in the RF group as compared to AL controls and that this increase is maintained after returning to ad libitum conditions (RF+AL), even after FAA is abolished.

Cell density estimates of c-FOS positive cells revealed a distinct pattern of neuronal activation as compared to AL controls for both the RF and RFAL groups (see **Figure 4**) for bar graphs and **Table 2** for accompanying ANOVA values). The following areas exhibited significant changes in the number of c-FOS expressing cells between groups: AcbC, AcbSH, Cg1, VTA, SCN, LH, PVN, VMH, ARC, DMH, DR, PB, NTS, LC, BNSTov, CeA, BLA, and DG. Planned comparison analysis revealed a significant increase in the number of c-FOS expressing cells in the RF group, as compared to AL negative controls, for all regions examined with only four exceptions: the PFC and VTA remained at AL levels (although the VTA does exhibit a statistical trend), while the SCN and PVN were lower than their respective AL counterparts (**Figure 4** and **Figure 5**). Interestingly, animals in the RFAL group still showed RF-like patterns of c-FOS expression in certain hypothalamic and extra-hypothalamic brain regions including the ARC, DMH, DR, PB and NTS (**Figure 4** and **Figure 6**). Expression of c-FOS in all other regions, however, returned to levels similar to or less than the AL group.

**Table 2:** ANOVA and planned comparison analysis of c-FOS cell density, circulating ghrelin levels, and stomach PER2 expression.

Region of Interest	ANOVA *F_(2,18)_; p*	RF vs AL *p* value	RFAL vs RF *p* value	RFAL vs AL *p* value
Arcuate Nucleus	**4.227; 0.041**	0.077	0.380	**0.015**
Basolateral Amygdala	**18.138; <0.001**	**0.002**	**<0.001**	0.056
Oval Nucleus of the Bed Nucleus of the Stria Terminalis	**10.102; 0.003**	**0.002**	**0.003**	0.853
Central Amygdala	**17.613; <0.001**	**<0.001**	**<0.001**	0.997
Cingulate Gyrus	**4.899; 0.023**	**0.011**	**0.027**	0.642
Dentate Gyrus	**17.692; <0.001**	**0.001**	**<0.001**	0.131
Dorsomedial Hypothalamus	**5.372; 0.022**	**0.022**	0.698	**0.011**
Dorsal Raphé	**5.182; 0.024**	**0.008**	0.334	0.053
Locus Coeruleus	**17.056; <0.001**	**0.001**	**<0.001**	0.541
Lateral Hypothalamus	**5.408; 0.021**	**0.010**	**0.022**	0.690
Nucleus Accumbens Core	3.108; 0.074	**0.048**	**0.046**	0.983
Nucleus Accumbens Shell	**9.508; 0.002**	**0.001**	**0.008**	0.271
Nucleus of the Tractus Solaris	**7.310; 0.008**	**0.004**	0.603	**0.011**
Parabrachial Nucleus	**33.189; <0.001**	**<0.001**	**0.013**	**<0.001**
Prefrontal Cortex	1.840; 0.193	0.114	0.121	0.977
Paraventricular Hypothalamic Nucleus	**5.127; 0.025**	0.061	**0.008**	0.300
Suprachiasmatic Nucleus	3.122; 0.081	**0.046**	**0.058**	0.904
Ventromedial Hypothalamus	**4.680; 0.031**	**0.016**	**0.030**	0.736
Ventral Tegmental Area	3.452; 0.058	0.094	**0.022**	0.451
Stomach PER2 ROD	**4.682; 0.031**	**0.023**	**0.020**	0.940
Acylated Ghrelin Conc.	**12.994; 0.001**	**0.001**	**<0.001**	0.824

The use of **Bold** highlights statistically significant differences at an alpha level of 0.05.

**Figure 7:**
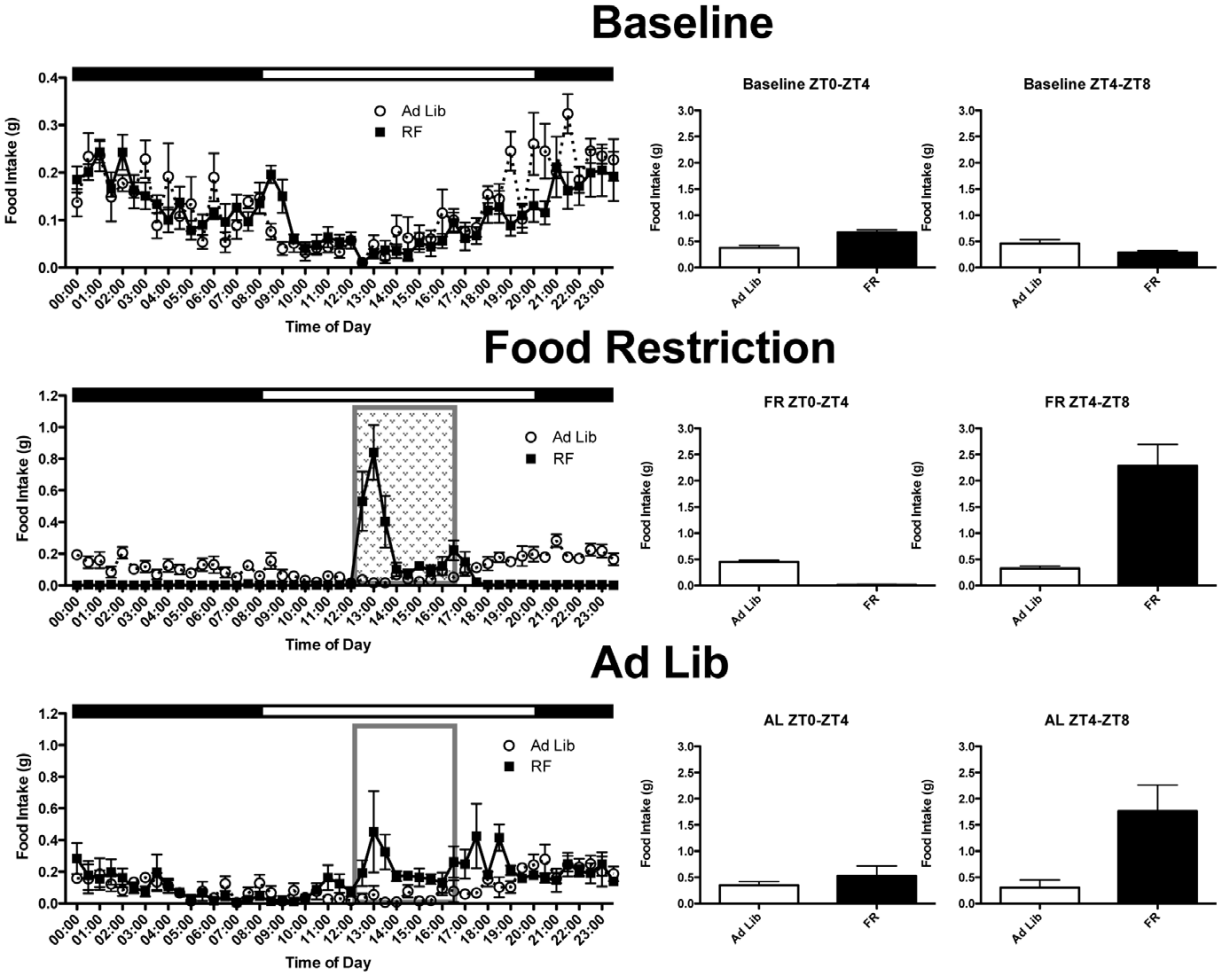
Rhythms of Food Consumption. Average waveforms representing food consumed over twenty-four hours by both AL and RF+AL groups under baseline, restricted feeding, and return to ad libitum conditions. Bar graphs to the right of each 24-hr profile show the amount of food consumed from lights on to the scheduled meal time (ZT0–4) and the amount of food consumed during the 4 hours of food availability (ZT4–8) for each condition.

A possible alternative explanation for the observation of elevated c-FOS expression in certain brain regions of RF mice, even after being placed back on *ad libitum* feeding conditions, is that they began eating during the morning after lights-on, during the period when they previously showed FAA. If animals had just consumed a large meal immediately prior to being sacrificed, satiety signals from the gut or circulating hormones could cause c-FOS activation in areas such as the ARC and NTS [33]. In order to address this possibility, separate groups of animals were run through a similar feeding protocol within metabolic chambers, where their food and water intake could be monitored continuously without disturbing the animals. This also had the benefit of avoiding a confounding timing cue i.e. the presence of a researcher at previous meal time, even in the absence of food removal or presentation after a return to *ad libitum* feeding. We found that animals returned to *ad libitum* feeding conditions did not eat more than the continuously AL animals during the period between lights on and the time of previous food presentation (ZT0–4), when FAA occurs under RF (**Figure 7**, middle panels). During this 4 hour period, AL animals ate an average of 0.35 g of chow (±0.071 g), which did not significantly differ from the food intake of RF+AL animals (T_[6]_ = 0.86, p>0.05). Interestingly, confirming previous reports from a similar feeding paradigm conducted in rats [34], RF+AL animals did continue to eat a daytime meal during the former food presentation period (ZT4–8; see **Figure 7**, bottom panels), consuming an average of 1.8 g of chow (±0.50 g), significantly more than AL animals (0.30 g of chow ±0.15 g, T_[6]_ = 2.8, p<0.05). This confirms that RF+AL animals did not eat a large meal prior to sacrifice, and instead supports the notion that the FEO continues to operate and entrain feeding, even in the absence of anticipatory behavior mediated by homeostatic and appetitive drives.

**Figure 8:**
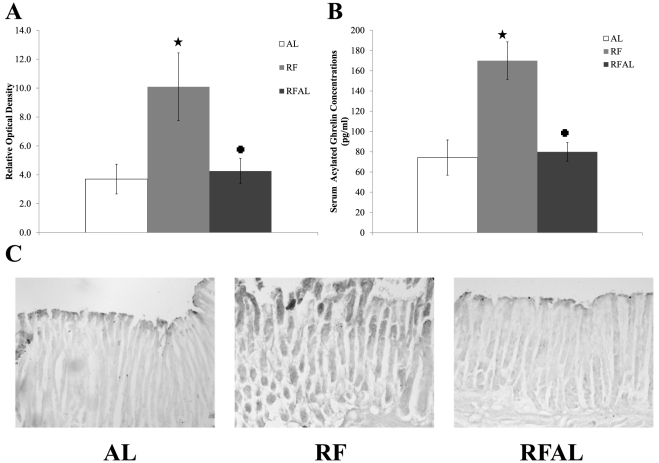
Stomach PER2 Immunocytochemistry and Plasma Acylated Ghrelin Assay. **A,** Average RODs of PER2 in the oxyntic mucosa of the stomach. **B,** Average concentrations of circulating active ghrelin. **C,** Representative images of PER2 staining. Note: stars indicates a significant difference from AL controls (*p*<0.05), crosses indicate significant difference from RF group (*p*<0.05).

As expected, analysis of plasma active ghrelin concentrations determined that there was a significant group difference (*F*_(2, 17)_ = 12.994, *p*<0.001). Planned comparison analysis revealed that while animals in the RF group developed elevated acylated ghrelin concentrations in anticipation of the scheduled meal (*p* = 0.001), this was no longer observable after a return to *ad libitum* feeding, i.e. the RFAL group did not significantly differ from AL controls (*p* = 0.824). This pattern was also accompanied by elevated levels of the clock protein PER2 within the stomach in the RF group (*p* = 0.023) and a return to AL levels after several days of *ad libitum* feeding (*p* = 0.940) (see **Figure 8**).

## Discussion

The current project examined whether neuronal activity stimulated by scheduled meals would persist in a sub-population of brain structures following the termination of the restricted feeding schedules, thereby highlighting the potential brain structure(s) housing the pacemaker of the food entrainable oscillator. Our results show that a number of hypothalamic and brain stem structures continue to show elevated c-FOS expression days after the termination of the restricted feeding schedule and return to *ad libitum* feeding.

Confirmation of food entrainment is generally tested by assessing the re-emergence of anticipatory behaviours in animals that are food deprived following a protracted *ad libitum* feeding period after entrainment by restricted feeding. We reasoned that if the FEO is a true pacemaker entrained by restricted feeding, then a neural representation of this timekeeping mechanism should persist, observable as persistent neuronal activity, following the restricted feeding schedule, much in the same way as the SCN continues to exhibit rhythmic neural activation after removal of the zeitgeber (*i.e.* under constant darkness conditions) [35]. This process would facilitate the appropriate timing of food anticipation the next time an animal encountered a shortage of food. Similarly, if the FEO is a true pacemaker then, once entrained, it must also produce self-sustained oscillations that are observable as a pattern of neural activity with stable peak-to-peak intervals. Comparing neuronal activation at the same circadian time in animals before, during, and after the restricted feeding schedule, was an attempt to sample the presumptive peak activation within these oscillating brain regions. We also reasoned that this neural representation should continue to be observed regardless of the metabolic state of the animal. This canonical pacemaker property, continued oscillations in the absence of a zeitgeber, was exploited in order to avoid c-FOS expression due to metabolic demand specifically in regions of the brain that are critically implicated in the homeostasis of food intake and metabolism. In examining these regions, we observed persistent c-FOS expression in select hypothalamic and caudal brainstem sites critical for nutrient sensing and energy balance, in the absence of any observable anticipatory locomotor activity. For instance, persistently increased c-FOS expression was detected in the DMH, ARC, DR, PB and NTS of mice in the RFAL group see (**Figure 4**). Individually, all of these regions have been investigated to determine whether they contain the FEO. Lesions destroying each of these nuclei individually, or targeted mutations to genes expressed by neurons in these regions, have been reported to transiently alter the ability of rats and mice to entrain to feeding schedules, and/or to show attenuated food anticipatory activity [36], [37]. To date, however, there is no single brain nucleus that has been pinpointed as both necessary and sufficient for the entrainment to feeding schedules [2]. In light of the findings presented here, this is not surprising, as all regions identified are sensitive to peripheral signals conveying information about metabolic state, and destruction of any of these individually may not be sufficient to prevent information about energy availability from reaching the brain and ultimately entraining biological rhythms to the time in which food is available.

Persistent c-FOS expression in the DMH further supports the importance of this hypothalamic region in the mechanisms underlying expression of FAA. It is still unclear whether the DMH is capable of intrinsic rhythmicity. Recent reports of circadian oscillations at the tissue level observed that rhythms of the DMH damped out very quickly *in vitro*, suggesting that it is not capable of self-sustained rhythms. However analysis at the single cell level suggests that it is a lack of synchrony between cells and not a lack of intrinsic rhythmicity which is the culprit. This strongly suggests that the DMH is capable of self-sustained oscillation at the cellular level, but that it requires an extrinsic coupling factor (possibly feeding-related) in order for continued coordination at the tissue level [38]. Neuronal activity in the DMH is increased in anticipation of scheduled meals, but it is also elevated around the previous meal time in rats that are placed in restricted feeding schedules where food is provided at random times during the day [39]. The rhythms of at least one clock gene, PER1, also tracks approximate meal presentation time, even in the absence of FAA [40], but peaks differentially according to meal duration [41]. This suggests an underlying sensitivity to metabolic demand, rather than a precisely timed mechanism tuned to food availability. Furthermore, inhibition of the SCN by the DMH has emerged as a mechanism whereby SCN neuronal activity is suppressed, allowing anticipatory locomotor activity to be expressed during a time when nocturnal animals are normally inactive. It was demonstrated that this suppression is necessary for the full expression of FAA [42] but a recent study suggests that this is only necessary if the meal presentation occurs outside of the normal period of arousal [43]. The DMH therefore represents one of several nuclei which disengage the SCN in order to allow the appropriate expression of FAA [5], while the observation of persistent activation within this region further suggests that it is integrated into a spatially distributed FEO pacemaker or, at the very least, that it is preferentially sensitive to FEO, rather than SCN, outputs.

Interestingly, the ARC, an area critical for energy balance [44], also remains active in animals previously exposed to a restricted feeding schedule (RF+AL group). Within the ARC, neurons release peptides critical for the regulation of metabolism, with α-MSH being important for increasing metabolic rate and decreasing food intake, and the agouti related peptide producing orexigenic responses and decreases in energy expenditure to promote obesity [45]. The effects of both these peptides are mediated by the same receptors, the MC3/4 receptors, located throughout the hypothalamus, and with very dense concentrations in the PVN, DMH and LH. Interestingly, mice with targeted deletions of these receptors show attenuated food anticipatory responses when exposed to scheduled meals [46], [47]. It is therefore possible that activation of the ARC during restricted feeding schedules is selective to neurons secreting agouti related peptide, and that these remain active in spite of the animals being returned to *ad lib* conditions. This idea is supported indirectly in our data by the observation of significantly elevated rebound feeding response once the mice are allowed to have free access to food (See **Figure 2b**) and it is also consistent with the role of Neuropeptide Y as an SCN suppressing signal when considering the extensive co-localization, and co-release, of these two neuropeptides within ARC neurons of animals under metabolic demand [48].

As expected, c-FOS expression was increased in other hypothalamic regions implicated in food intake and metabolism including the VMH and LH. However this activation was likely due to metabolic demand and/or the result of outputs signals of the FEO, or release from inhibitory inputs from the SCN, as it was only observable during RF. In contrast, the SCN and PVN of mice under the restricted feeding schedule showed decreased c-FOS expression, with neuronal activity in these areas being restored to baseline levels by a return to *ad libitum* feeding (**Figure 4** and **Figure 6**). This is in agreement with similar observations in rats during FAA, at least for the SCN [39], [42]. In terms of both evolutionary adaptation, as well as economy of function, the necessity for SCN-uncoupling during FAA is not surprising, and has been proposed as a mechanism facilitating entrainment to feeding schedules [2], [5], [43], [49], [50]. A recent investigation has implicated the DMH in the suppression of SCN activity, specifically within the ventrolateral core, and this occurs via direct axonal projections from food sensitive neurons within this nucleus [51].

While c-FOS activation of the PVN was not expected, the observed decrease in c-FOS-IR in RF animals has not been widely reported, but was found in rats simply fasted for 64 hours [52]. That we observe this decrease may be due to the power of the stereological approach but may also be related to the extreme fragility of mice under metabolic demand. Inhibition of activity within the PVN in food-restricted mice may also be related to increases in activity in other brain regions including the DMH and the BNSTov. Like the SCN, the PVN is directly innervated by both glutamatergic and GABAergic projections from the DMH [53], [54]. Interestingly, even though both excitatory and inhibitory cells are found in the DMH, lesions of the DMH lead to increased hypothalamic-pituitary axis responses to stressors [55] and pharmacological stimulation of DMH neurons produces inhibitory post-synaptic potentials in neuroendocrine cells of the PVN [56]. This is consistent with a strictly inhibitory role of the DMH on the PVN but implies an ability of the DMH outputs to switch from inhibitory to excitatory depending on the intrinsic rhythms of the animal, nocturnal or diurnal, as has been recently proposed [57]. Similarly the PVN is heavily innervated by the BNSTov [58] via GABAergic projections [59], [60]. Ablation of the posterior BNST, where the oval nucleus is situated, increases c-FOS expression and CRH release within the PVN, indicating that the BNSTov plays a significant role in the suppression of these two mechanisms within the neuroendocrine system [61]. It is proposed by Cullinan et al. [60] that this represents a braking mechanism by which afferents of the forebrain limbic system can modulate the stress response, and this agrees with the results achieved in this experiment. While acute food deprivation has been shown to increase the stress response, repeated exposure to a predictable meal schedule should ideally decrease the stress response due to an ability to anticipate the near-future availability of food and the BNSTov likely plays a major role in the neural mechanism responsible.

Neurons in the NTS and PB are important targets for both circulating metabolic hormones, as well as visceral signals conveyed through the vagus nerve [62], [63], [64]. Because of this, both of these structures have been suspected in participating in the processes underlying food entrainment, although lesions to PB attenuated FAA but did not appreciable affect entrainment [65]. Similarly vagal deafferentation has no effect on FAA [66]. Yet, persistent activation of these brain regions may reflect the action of endocrine and/or afferent visceral signals that continues to act in spite of *ad lib* food availability. Alternatively, continued activation of these brain stem nuclei may be tied to food consumed just prior to the animals being sacrificed. Indeed, signals like leptin and cholecystokinin (CCK) act directly and synergistically to stimulate these brain stem regions and induce satiety [67]. In order to address this, our second experiment examined patterns of food consumption in animals under the various schedules. We observed continued daytime feeding behaviour, even after a return to *ad libitum* food availability (see **Figure 7**), however this occurred entirely during the time of previous meal presentation (ZT4–8) indicating that our RF+AL mice would not have consumed a meal prior to sacrifice and that the c-FOS expression observed is most likely a neural representation maintained by exposure to a previous restricted feeding schedule. Furthermore, if the animals had indeed eaten prior to sacrifice we would expect that mice in the RF+AL condition would have much greater c-FOS expression in the NTS and PB as a result of that meal compared to mice in either the AL or RF group [68]. It is important to add that visual inspection of the stomach at the time of sacrifice provided no clear indication that the mice in the RF+AL group had consumed more food than animals in the other groups just prior to sacrifice, again supporting the idea that persistent c-FOS expression in the NTS and PB is related to the previous restricted feeding schedule and not to recent caloric intake. Critically, this hypothesis is further supported by the return of clock protein PER2 expression in the oxyntic mucosa of the stomach - putatively linked to rhythmic food intake [69] - to ad libitum levels after only three days. This last finding fits with PER1 transcription timing from a previous *in vitro* experiment in which *per1:luciferase* rhythms from rat stomachs were entrained to RF schedules and similarly returned to their original phase within days of a return to *ad libitum* feeding [70].

Observations of increased activation in the brainstem in anticipation of a meal, as well as the continued activation of the NTS and PB after a return to *ad libitum* feeding, conflicts with the findings of two other groups conducted in rats. In the first study [28], the authors maintain that there was no anticipatory c-FOS expression observed in the NTS, PB or the Area Postrema as compared to *ad libitum* fed controls, and activation was only observed after the feeding bout. While it was true that there was increased activation (almost four-fold) observed after feeding in all three of these brain stem nuclei related to visceral signals of satiety, a closer inspection of their results prior to the expected presentation of food showed apparent increases in activation of the medial NTS and medial PB during food entrainment and the medial PB during fasting after entrainment. These time points and nuclei correspond directly with this study. These changes appear statistically significant, although this was not tested in their study, but were much smaller in magnitude to the drastically increased activation observed after feeding. This small change may represent a subpopulation of cells within these regions and if this is the case our stereological analysis would be much more likely to resolve this for the following reason: Angeles-Castellanos et al., used a single 40 µm brain slice for cell counting in each region of interest. By comparison, the present study performed a stereological investigation of each region, creating an estimate of the activation over the entire extent of each ROI and therefore had greater power to detect small but significant changes both in anticipation of the meal (RF) and at the time of previous anticipation (RFAL). In the second study [71], Poulin et al. observed no anticipatory increase of c-FOS mRNA within the NTS. The reasons for this may be two-fold. Measurements of optical density from *in situ* hybridization reflects a direct correlation of the *cfos* mRNA levels. In contrast, our method of counting positively labeled c-FOS protein expressing cells does not correlate directly with the amount of c-FOS protein expression. The number of immunopositive cells is determined by the specific conditions and method used to visualize immunoreactivity, including the threshold of detection of our antibody at the concentration used, the amount of time the DAB reaction was developed, etc. From this, a binary analysis is then performed whereby the observer answers a simple question. Is protein visible compared to background, i.e. can a nucleus be visualized. If the answer is yes, the cell is counted. This creates a situation whereby even small changes in the protein level of each cell can quite significantly alter the total number of c-FOS positive cells. A more direct measurement of the relative amount of c-FOS protein can be done using a Western blot, but this does not allow for precise anatomical localization of the protein. Secondly, while protein levels typically follow up-regulation of mRNA, it is more difficult to compare timing of expression, as mRNA translation and protein transcription can be differentially regulated. There are also post-translational events which can regulate protein expression independently of transcription as has been previously shown for c-FOS [72]. Any of these could account for a discrepancy between the results suggested by *in situ* hybridization vs. immunocytochemisry.

Finally, the dorsal raphé nucleus showed increased c-FOS expression in anticipation of the scheduled meal and this activity persisted in animals that were allowed to return to *ad lib* feeding. While there is little data linking the raphé nuclei with the generation of food entrainment, this region has been implicated as a potential photic and non-photic modulator of circadian rhythms of activity via it projections to the SCN [73], [74], [75], [76], [77], [78]. Interestingly, the raphé of rats exposed to restricted feeding schedules (food available for two hours during the day for 15 days early in the light phase of the light dark cycle) show increased c-FOS expression during food search (i.e. prior to meal presentation) in comparison with ad lib controls and animals on the same schedule that are allowed to eat until satiation [27]. Our study confirms these data in mice, and shows that the raphé continues to be active in anticipation of a previously scheduled meal.

An increase in c-FOS across brain regions along mesolimbic and mesostriatal dopaminergic reward pathways was observed in anticipation of a scheduled meal. These data confirms previous reports implicating reward processes in food anticipation [79], [80], [81], [82], [83]. However, neuronal activity in these regions return to baseline, indicating that the effects of the restricted feeding schedule on the reward circuits do not persist beyond the restriction paradigm. Similarly, brain circuit associated with arousal (LH, LC) as well as those mediating emotional responses (BNSTov, hippocampus, amygdala) follow the same pattern. From this we infer either a diminished or secondary role of these other nuclei in the maintenance of food availability timekeeping while still supporting their necessity for the appropriate expression of food anticipatory behaviours and physiology.

Finally, a similar same pattern was observed for plasma concentration of acylated ghrelin, consistent with a return of PER2 levels in the stomach to baseline. It has previously been suggested that ghrelin may be either a tuning signal from the periphery [84] or a coupling mechanism between pacemaking components of the FEO in the stomach and brain [69]. The present findings confirm that levels of the active form of ghrelin does not persist after a return to ad libitum feeding, nor does the increased level of PER2 in the stomach, thus the circadian oscillators in the stomach are not critically involved in the pacemaking mechanism of the FEO. Therefore, ghrelin signaling may act as a tuning mechanism, and possibly as one of many zeitgebers, conveying information about food availability from the periphery to numerous brain sites sensitive its signal—including many of those investigated in this study—in order to promote neural activation necessary for the appropriate expression of FAA.

In the present study, we have revealed brain areas which remain active in mice previously exposed to a restricted feeding schedule and then allowed free access to food, in comparison to ad lib fed mice (negative controls) and in mice that remained under the restricted feeding schedule (positive controls). Our findings, while not comprehensive, confirm that a number of hypothalamic and brain stem regions persistently activate at the time of prior meal anticipation including the DMH, ARC, DR, PB, and NTS. Furthermore this persistent neuronal activity was not ubiquitous; suggesting that any region with continued activation at the time of the previous scheduled meal may be directly involved in maintaining a neural representation necessary for timekeeping of previous food availability. A number of groups have proposed that the FEO may indeed be a network of interconnected brain structures [29], [71], [85], [86], [87] and this proposition has recently received a thorough review [14]. It is our belief that this is indeed the case, and while a correlative study such as the one presented here is incapable of incontrovertibly demonstrating that all structures identified are both necessary and sufficient for the pacemaker function of the FEO, both the number of well documented interconnections between the regions identified here, as well as the sheer number of regions identified as persistently active further suggests that this is the case. Our assertion is also based on a principle assumption of this study: that after a return to ad libitum conditions ONLY the regions that are necessary for timekeeping would remain preferentially activated. We also assert that there is no conceivable reason why either upstream effectors (as there is no food, peripheral or other cues expected/presented at this timepoint) nor downstream affectors (which are reset to normal, SCN driven, light/activity/food consumption/body temperature rhythms for appropriate control of behaviour and physiology under *ad libitum* conditions) should be influencing the levels of c-FOS expression in regions not critically involved in the timekeeping mechanism of the FEO.

This study thereby contributes to the search for a neural system tasked with the circadian entrainment to daily meals (the FEO) by narrowing down a large list of previously proposed candidates and presents an experimental paradigm useful for future studies in which it will be necessary to dissociate ancillary activation due to the various homeostatic, appetitive, and affective components driving FAA.
